# Changes in the microbiological, physicochemical properties of Chinese traditional fermented Suan rou at ripening fermentation

**DOI:** 10.1002/fsn3.2095

**Published:** 2021-09-04

**Authors:** Xuefeng Zeng, Ju Meng, Wei Zhang, Laping He, Li Deng, Chun Ye

**Affiliations:** ^1^ School of Liquor and Food Engineering Guizhou University Guiyang China; ^2^ Guizhou Provincial Key Laboratory of Agricultural and Animal Products Storage and Processing Guiyang China; ^3^ Key Laboratory of Animal Genetics, Breeding and Reproduction in the Plateau Mountainous Region Ministry of Education Guiyang China; ^4^ College of Food Science and Engineering Wuhan Polytechnic University Wuhan China

**Keywords:** microbiological, organic acids, sensory, suan rou

## Abstract

This study characterized the changes in the microbiological, physicochemical properties of Suan rou during fermentation via three different techniques (Technique A is a traditional production process. Based on technique A, technique B adds a total of 200 g of sucrose to the thinly sliced meat, and technique C changes the amount of salt in the thinly sliced meat to 200 grams.). Compared to batch A, the samples from batches B and C featured more rapid reduction in pH and generated more TA. Myofibrillar proteins in batches B and C showed higher degradation rate, and several low‐molecular‐weight metabolites were determined on the basis of sodium dodecyl sulfate polyacrylamide gel electrophoresis (SDS‐PAGE) gel lanes. The contents of thiobarbituric acid (TBARS) and total volatile base nitrogen (TVB‐N) and the growth of spoilage bacteria and pathogens were suppressed in the three batches. A relatively compatible acid–salinity proportion was presented in the Suan rou of batches A and B compared with that of batch C. The results show that the Suan rou made by B technology was more palatable acid flavor and abundant nutrition.

## Graphical abstract

Suan rou is a uniquely tasty and highly safe meat product made by traditional natural fermentation and deeply favored by local consumers. This study characterized the changes in the microbiological and physicochemical properties of Suan rou during fermentation via three different techniques (i.e., A, B, and C). These results show that the Suan rou made by B technology had the most palatable acid flavor and abundant nutrition among the three different batches of Suan rou.

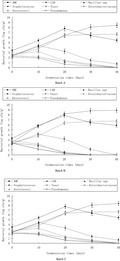

## 1 INTRODUCTION

Suan rou is a traditional, uniquely tasting, highly safe meat product, made by traditional natural fermentation, widespread in ethnic minority areas, such as Guizhou, Yunnan, and Hunan, China (Dai, 2009). Streaky pork is the usual raw material used in areas that consume Suan rou. The pork is sliced in a certain size and mixed with salt in a certain proportion. The pork is then evenly mixed with glutinous rice flour after low‐temperature curing. Next, spices and chili powder are added to the pork, which is then placed in a sealed ceramic jar and fermented for 1–2 months. In this manner, the prepared Suan rou becomes mildly acidic and fatty but not greasy; it also has a long shelf‐life and low cholesterol and can be kept for several years (Dai, 2009). In the meat industry, the production of sour meat is novel, with low pH and unique sourness. At present, it is only produced in a few areas, has broad exploration potential, and is a new type of meat product.

High numbers of lactic acid bacteria (LAB), coagulase‐negative staphylococci, yeast, and mold often exist in traditionally fermented meat products, in which LAB can reduce the pH of the product by generating lactic acid, thus making the product appropriately sour and effectively safe (Zeng et al., 2013a). *Staphylococcus*, yeast, and mold play important roles in flavor and color enhancement (Nie et al., 2014). Given that pH is reduced close to the isoelectric point of proteins in fermented meat products, a portion of the fermented meat product will be dehydrated, resulting in the reduced water activity (aw) of the products (Yoo et al., 2007). Textual parameters, such as hardness, springiness, and cohesiveness, will change accordingly (Weiss et al., 2010). The inherent endogenous enzymes and portions of the LAB, yeast, and *Staphylococcus* in naturally fermented meat products exhibit certain proteinase and lipase activities; these enzymes can degrade macromolecules, such as proteins and fats, in meat products and form corresponding small molecular substances, such as polypeptides, free amino acids (FAA), and free fatty acids (FFAs) (Yang et al., 2015). Furthermore, the reaction products from autoxidation of Lipids, which involves inanimate objects or spices, and flavors generated by carbohydrates are also produced in the natural fermentation of meat products (Spaziani et al., 2009). All of these small molecular compounds can notably enhance the palatability and flavor of fermented meat products, thus improving their quality.

Dominant microbe production and complicated physicochemical and substance flavor changes also exist in the natural fermentation of Suan rou. The corresponding dominant microbes and metabolites of biochemical reactions will influence the final sensory property of Suan rou. A more common is the existence of three different formulations of Suan rou in western Hunan, in which the technical A is to add salt, pepper, star anise, cinnamon powder and ginger slices to the meat slices. On the basis of technical A, technical B increases sucrose and corn flour, and technical C is to reduce salt content and add corn flour. Sucrose, as an energy source and a sweetener in a product, has an effect on the physical and chemical parameters of the product. Salt content also affects the diversity of microorganisms and the flavor of the product. Therefore, there will be some quality differences in the Suan rou produced by the three technologies. However, there are few studies on the quality differences of these three main Suan rou formulations. For this reason, this study aimed to investigate the microbiological, physicochemical, texture, and sensory characteristics of three different techniques of Suan rou during fermentation. By comparing the microbiological, physicochemical, texture, and sensory characteristics of different formulations of Suan rou, better industrial production formulations can be obtained.

## 2 MATERIALS AND METHODS

### 2.1 Sample Preparation

Three different techniques (A, B, and C) were used in this experiment for the production of low‐salt Suan rou in western Hunan, China. Technique A was performed as follows. Fresh streaky pork (3–5 cm‐width and 3–4 cm‐thick) was sliced. For every 10 kg streaky pork, 400 g salt, 10 g Chinese prickly ash, 10 g *Illicium verum*, 10 g Chinese cinnamon, 100 g dry chili powder, and 400 g ginger slice were added. The materials were mixed, refrigerated, and cured for 24 hr. Then, 75% cured streaky pork (2.6 kg corn flour, 100 g salt, 2.5 g Chinese prickly ash, 2.5 g *Illicium verum*, and 2.5 g Chinese cinnamon) and stir‐fried 25% corn flour (COFCO, Shanghai, China) were evenly mixed and then placed in a jar or glass bottle. Cover the cap. After the edge of the jar was sealed with water, the bottleneck was plugged with straws or corn bran. Suan rou can be formed after 30 days of natural fermentation. Technique B was conducted as follows. A total of 200 g saccharose were added to the sliced meat based on technique A. Technique C was applied as follows. The amounts of salt in the sliced meat were changed to 200 g based on Technique A. During fermentation, samples from the three different low‐salt Suan rou batches were collected every five days for relevant microbiological and physicochemical analysis.

### 2.2 Microbiological analysis

Approximately 25 g Suan rou sample was obtained from each group, ground, homogenized (UltraTurrax homogenizer, IKA Labortechnik, Selangor, Malaysia), and then added into 225 ml sterile NaCl water (0.9%). After step‐by‐step gradient dilution, appropriate decimal dilutions of the three sample solutions were selected. Exactly 0.1 ml solution was then obtained from each sample and poured into different selective media for cultivation to quantify different microorganisms. Three parallel tests were conducted for each sample solution. Total viable counts were grown aerobically for 3 days at 30°C by using PCA (Oxoid, Basingstoke, England). LAB were cultivated for 2–3 days at 30°C in MRS agar. *Staphylococcus* were cultivated for 2 days at 37°C in MSA. Yeast was cultivated for 3–4 days at 25°C in PDA, and Enterobacteriaceae were cultivated for 1 day at 37°C inVRBDA. *Bacillus* spp. were cultured in DTA at 35°C for 2 days and counted, whereas *Bacillus* spore formers were determined by using 10‐fold dilutions of *Bacillus* spp. incubated in DTA for 2 days at 35°C after pasteurization for 10 min at 80°C. The *Pseudomonas* incubated in *Pseudomonas*–*Aeromonas* selective agar at 26°C for 3 days were counted. Enterococci incubated in KAA at 42°C for 1 day were determined.

The dominant bacteria were isolated from the highest dilution gradient of Suan rou samples at different fermentation stages and then purified in the aforementioned various media. The DNAs of LAB and *Staphylococcus* were extracted using the method of Benito (Benito et al., 2008), and the specific types of bacteria were analyzed by 16S rRNA gene sequencing. Yeast DNA was extracted using the method of (Kurtzman & Robnett, 1998), and the types of bacteria were analyzed by 26 rRNA gene sequencing.

### 2.3 Proximate chemical analyses

Protein content was determined in accordance with the Kjeldahl method (AOAC, 1997). A 6.25 factor was used for the conversion of nitrogen content to crude protein. Sarcoplasmic and myofibrillar proteins were extracted according to the methods of Mauriello et al. (2002), and the protein concentrations were measured as described by Lowry et al. (1951).

Crude fat content was determined by Soxhlet method as described in AOAC (1997). Cholesterol levels were determined by enzyme catalytic spectrophotometry according to the manufacturer's instructions. The levels of FFAs were assayed using the method of ACS‐ACOD as described by the manufacturer's instructions (Beijing nine Strong Biotechnologies, Beijing, China).

Aw was measured using a digital aw meter (Rotronic Hygroskop DT, Zurich, Switzerland) after stabilization at 25°C for 30 min. Salt content was analyzed using an AOAC procedure (1997).

The pH was determined according to the method of Wang (2000). Approximately 10 g sample was weighed and added with 90 ml deionized water. The pH was determined using a digital pH meter (Mettler Toledo 320‐s, Shanghai, China) after the sample was homogenized at 8,000 g for 1 min.

Total acidity (TA) was analyzed in accordance with the AOAC guidelines (1997). Exactly 5 g sample was weighed and added with a small amount of distilled water without carbon dioxide. The sample was homogenized at 8,000 g for 1 min and then transferred to a 250 ml volumetric flask. After heating in water bath at 75°C–80°C for 30 min, the sample was cooled until constant volume and filtered (initial liquid was discarded). The obtained filtrate was titrated with 0.1 mol/L standard NaOH. Finally, the TA was expressed by % lactic acid.

Organic acids were determined according to the method of (Riebroy et al., 2004). Approximately 15 g sample was added with 35 ml distilled water, homogenized at 8,000 g for 1 min, and then centrifuged (Sigma Laborzentrifugen, Model 4K15, Osterode, Germany) at 4,500 g for 15 min. 0.9 ml of supernatant was obtained and evenly mixed with 0.1 ml 5% (w/v) internal‐standard butyric acid. The mixture was added with 0.5 M perchloric acid for 5 min. The mixture was then centrifuged at room temperature and 12,000 g for 30 min to remove the protein residue. The supernatant was filtered using 0.45 µm membrane (Minisart RC‐15, Satorious, Goettingen, Germany) and injected into the high‐performance liquid chromatograph (HPLC) (Agilent1100, USA) with an Ecomsil C18 (4.6 mm × 250 mm). The components and contents of different organic acids in the samples were identified on the basis of the peak area and retention time. The conditions of HPLC were as follows: mobile phase: methyl alcohol:water:phosphoric acid = 5:95:0.05 (v/v), gradient elute. Chromatographic conditions were as follows: 30°C column temperature; 0.8 ml/min flow rate; 210 nm photodiode wavelength.

### 2.4 Sodium dodecyl sulfate polyacrylamide gel electrophoresis (SDS‐PAGE)

Approximately 3 g samples were homogenized (Ultra Turrax homogenizer, IKA Labortechnik, Selangor, Malaysia) with 27 ml solubilizing agent (Tris‐HCl 8.0 buffer, 2% SDS, 8 M urea, and 2% β‐mercaptoethanol). The homogenate was then heated at 85°C for 60 min and then centrifuged at 10,000 g at room temperature for 15 min. Protein concentration was estimated using the Folin–phenol reagent method proposed by Lowry et al. (1951). The supernatant was mixed with 1:1 (v//v) SDS‐PAGE sample buffer (0.125 M Tris‐HCl, pH 6.8, 4% SDS, 10% glycerol, and 0.005% bromophenol blue). SDS‐PAGE was assessed as described by Laemmli (1970) and implemented in a vertical gel electrophoresis unit (Mini‐Protean‐3 Cell, Bio‐Rad, Richmond, CA, USA) by using a 10% separating gel with 4% stacking gel; 10 µl mixture was loaded in each well. Electrophoresis was employed at constant voltage of 100 V with current limit set to 90 mA for 45 min. After electrophoresis, gel staining and destaining were performed as described by Landeta et al. (2013).

### 2.5 FAA Determination

The FAA content of Suan rou was determined according to the method of (Aristoy & Toldra, 1991). Approximately 5 g Suan rou was precisely weighed, and 25 ml of 5% perchloric acid was added. The mixture was homogenized for 5 min and transferred to a 25 ml volumetric flask, and 5% perchloric acid was used to reach a constant volume of 25 ml. The solution was allowed to settle for 2 hr at 4°C. The supernatant was filtered by a double‐layer filter paper. Approximately 5 ml filtrate was centrifuged at 10,000 g for 5 min. FAA content was determined using the HPLC. Peak identification and quantification were completed by determining the retention time and recoveries of FAA standards (Sigma Chemical, St Louis, MO, USA).

### 2.6 Determination of FA

Determination of FFA was performed according to the methods of (Bligh & Dyer, 1959). Exactly 5 g sample was weighed, and 10 ml concentrated hydrochloric acid and 20 ml methyl alcohol were added and homogenized for 2 min. Approximately 10 ml concentrated hydrochloric acid was then added and sufficiently homogenized for 2 min. The mixture was homogenized for another 2 min after the addition of 18 ml low‐concentrated salt solution (0.88%). The mixed liquid was then layered after 10 min of centrifugation at 2000 r/s, and the supernatant liquid was collected into a separating funnel. Approximately 20 ml of 10% methyl alcohol and hydrochloric acid solution were added and homogenized for 2 min. Hydrochloric acid was mixed with the first extracted solution and then removed using rotary evaporation. The solution was dried at 104°C for 1 hr, and fat was weighed. Methyl esterification was conducted according to the methods of (Leseigneur‐Meynier & Gandemer, 1991). Fat concentration was determined by gas chromatography under the following conditions.

Detection conditions: quartz capillary chromatographic column: 30 m × 0.32 mm; flame‐ionization detection and carrier gas: helium 3 ml/min.

Column temperature: 120°C for 3 min, 190°C at 10°C/min, 220°C at 2°C/min, 220°C for 15 min.

Gasification: 250°C, detection: 250°C, and split ratio: 1:8

### 2.7 Total Volatile Basic *N* (TVB‐N) Determination

The TVB‐N content was determined by the microdiffusion method of Conway (Hughes et al., 2002).

### 2.8 TBARS Determination

TBARS shall be determined by referring to the methods of Buege and Aust (1978). A total of 5 g sample were added into 25 ml of TBARS solution (0.375% TBA, 15% TCA, 0.25 mol/L HCl) for homogenization and then heated in a water bath for 10 min under 95ºC to 100ºC until the solution turned into pink. Afterward, the solution was cooled and then centrifuged for 25 min under 5,500 g. Finally, an appropriate amount of supernatant was taken and placed at the position of 532nm of a spectrophotometer to determine the absorbance. The results are shown as mg MDA/kg.

### 2.9 Textual Determination

The samples were cut into slices with the dimension of 10mm × 30 mm, and then the hardness, elasticity, and chewiness of the fermented sour fish were detected by a texture analyzer (TA‐XT2i texture analyzer, Stable Micro Systems Company of Britain). The testing conditions are as follows: probe diameter: 50 mm; compression speed: 5 mm/s; compression deformation: 50%; time slot for second compression: 1 s; surface induction: 99 g; primary force: 30 g. The determination result was processed by Texture Expert (V1.0).

### 2.10 Sensory analysis

Because the taste of products of different processes is quite different, it is necessary to pick from three different processes of Suan rou to meet consumer preferences. The differences among the Suan rou made by traditionally natural fermentation were compared by preference analysis. Since we are a food college, the teachers and students have a certain tasting ability. So a total of 50 teachers and postgraduates in food professional in the laboratory were selected to conduct sensory evaluation of Suan rou. The fermented Suan rou was sliced into 5 mm thickness, placed in the tasting plates, and then numbered randomly. The appearance, flavor, color, acidity, salinity, texture, and general acceptability of the samples were scored by a 9‐point system: 1 for the worst, 5 for moderate, and 9 for the best (Paglarini et al., 2020).

### 2.11 Statistical analysis

All experiments were performed in three replicates, and the mean values were calculated. Statistical analysis was performed using the SPSS statistical program (Version 22.0) for Windows (SPSS Inc., Chicago, IL). ANOVA was used to determine the differences between sample batches, and the mean values were evaluated using Duncan's multiple range or least‐square difference test (*p* < .05). Person correlation coefficient was used in bilateral correlation analysis. *p* < .01 indicates highly significant difference, and *p* < .05 indicates significant difference.

## 3 RESULTS AND DISCUSSION

### 3.1 Microbiological Analysis

**FIGURE 1:**
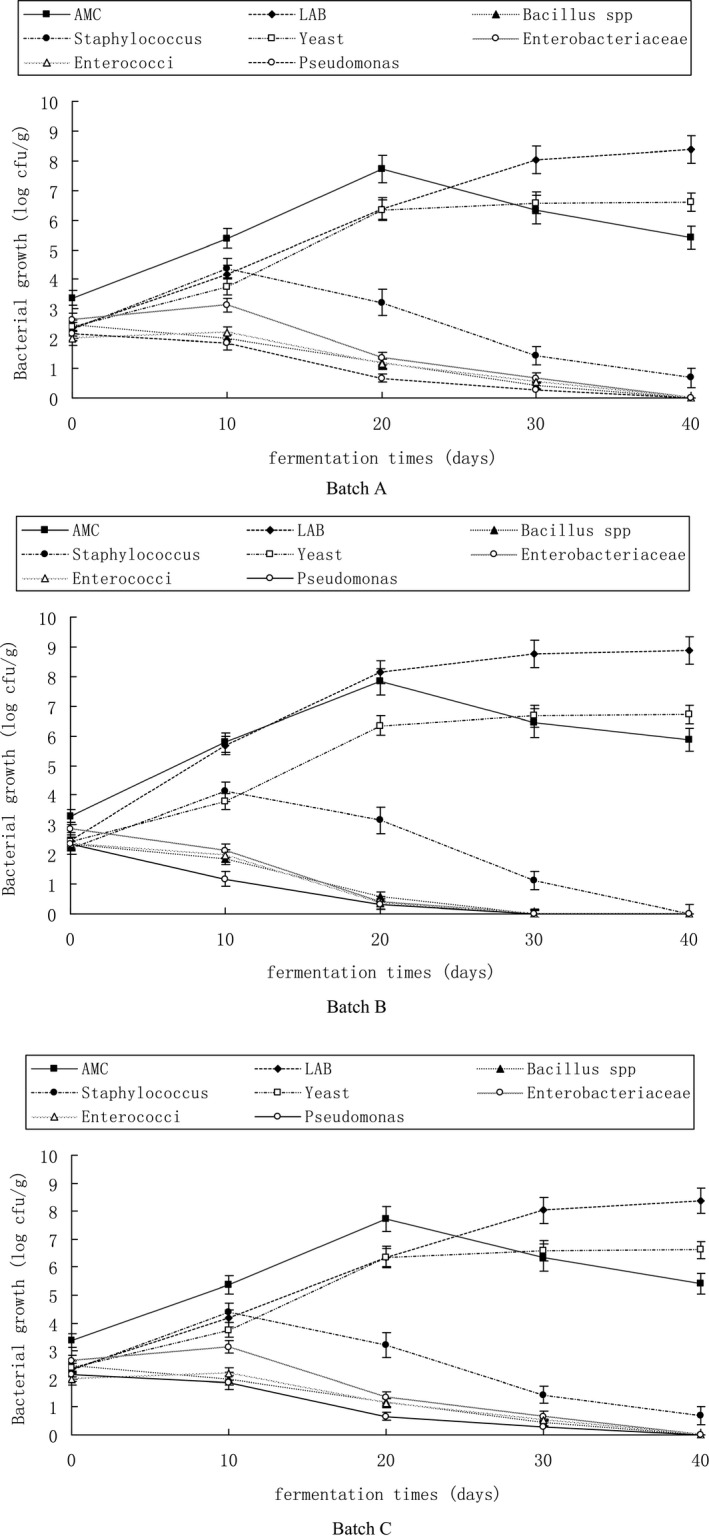
growth of bacteria species during the spontaneous fermentations of Suan rou. AMC, aerobic mesophilic count; LAB, lactic acid bacteria. Closed square, AMC; Closed rhombus, LAB; Closed circular, Staphylococcus; Closed triangle, Bacillus spp; opened square, Yeast; Opened circular, Enterobacteriaceae; Opened rhombus, enterococci; Opened triangle, pseudomonas.

Figure 1 shows the changes in the numbers of different microorganisms in Suan rou fermentation. The levels of aerobic mesophiles in the three samples gradually increased from 3.24 –3.37 log cfu/g to 7.72–7.84 log cfu/g in 20 days. The numbers then gradually decreased to 5.41–5.86 log cfu/g at the end of fermentation. The results were similar to those reported by Zeng et al. (2017) on traditional Chinese Suan yu. According to the research of Anihouvi et al.(2013), the increased amounts of aerobic mesophile in the initial fermentation may be due to the contamination of raw materials and curing, resulting in an increase in the number of aerobic or facultative anaerobic bacteria. With prolonged fermentation, LAB reached a relatively high level, and pH decreased to relatively low level, suppressing the growth of aerobic mesophiles. In addition, oxygen was depleted in the jar with fermentation time. Therefore, aerobic mesophile levels gradually reduced at the end of fermentation.

Figure 1 presents that a lower growth trend of LAB in batch A samples compared with that in batches B and C during fermentation. The number of LAB increased from 2.31–2.46 log cfu/g to 8.15–8.17 log cfu/g in 20 days. The levels were maintained until the end of fermentation. The number of LAB in batch A samples reached 7.29 ± 0.31 log cfu/g after 20 days. The number of yeasts also showed a marked increase in the three sample groups during fermentation. According to the research results of (Hu et al., 2008), the nutrients required for microbial reproduction can be provided if saccharose is added to the fermented meat product, therefore promoting the reproduction of LAB and yeast; the reproduction of part of the LAB can also be promoted if 2% salt is added into the fermented meat product, whereas the reproduction of most yeasts remains uninfluenced. As a result, in our research, samples in batches B (with 4% saccharose added) and C (with 2% salt reduced compared with batch A) showed faster reproduction effects on LAB and yeast. The strong correlation of the reproduction of LAB and yeast reproduction occurred in the three sample groups during the fermentation of Suan rou (*p* < .01, *r* = .969; *p* < .01, *r* = .983; *p* < .01, *r* = .992). The LAB with maximum valid dilute through separation in the three groups of Suan rou samples were identified as *Lactobacillus plantarum* and *Pediococcus pentosaceus* by the identification method of 16S rRNA molecular biology. The yeast with maximum valid dilute double in the Suan rou, as determined through separation, was identified as *Saccharomyces cerevisiae* by the identification method of 26S rRNA molecular biology. This result indicates that *L. plantarum* and *P. pentosaceus* can sufficiently utilize nutrient substances in the meat and were thus dominant during processing and fermentation of Suan rou. The color, flavor, and texture of the product can be improved through the acid and bacteriocin generated during fermentation; the maturity of the product can be accelerated, and the growth and reproduction of other putrefying and pathogenic bacteria can be restrained, thus improving the quality and ensuring the safety of the product (Lorenzo et al., 2012). Yeast can utilize the metabolites of LAB to proliferate quickly in the acidic environment generated by LAB and simultaneously secrete the metabolites required by LAB growth; thus, these organisms can promote the reproduction and growth of each other (Zeng et al., 2013b).

The number of *Staphylococcus* in Suan rou reached 2.22 –2.57 log cfu.g^–1^ at the initial period of fermentation. However, with extension of fermentation time, the number of *Staphylococcus* in batches B and C increased to 4.12 ± 0.13and 4.13 ± 0.12 log cfu.g^–1^ on the 10th day of fermentation, respectively. With extension of fermentation period, the number of *Staphylococcus* slowly decreased, and the numbers in the sample groups decreased to 1.12 ± 0.04 and 0.97 ± 0.03 log cfu.g^–1^ on the 30th day of fermentation. In the batch A samples, the number of *Staphylococcus* increased to 4.37 ± 0.13 log cfu.g^–1^ on the 10th day of fermentation. With the further extension of fermentation time, the number of *Staphylococcus* in batch A samples decreased to 1.43 ± 0.05 log cfu.g^–1^ on the 30th day of fermentation. This result indicates that the growth of *Staphylococcus* was evidently restrained in the middle and later periods of fermentation. Similar theories can also be obtained in the analysis of bacterial flora distribution and bacterial characteristics of traditional Chinese Suan yu, suggesting that *Staphylococcus* is notably restrained in acidic conditions (Zeng et al., 2013b). In a research on the dominant *Staphylococcus* in salami, (Bonomo et al., 2009), the staphylococci at pH under 4.5 could hardly grow and reproduce because the acidic environment had restrained their growth, whereas the fermentation of Suan rou consistently occurs in anerobic status. For this reason, we speculated that the anerobic conditions of fermentation and the acidic environment in the middle and later periods of fermentation have restrained the growth and reproduction of staphylococci in Suan rou. The generation of high amounts of LAB in the samples of batches B and C resulted in the rapid generation speed of lactic acid, thus further promoting the notable inhibitory effect on staphylococci in fermentation. The staphylococci in Suan rou with maximum valid dilute double through separation were identified as *Staphylococcus xylosus* and *Staphylococcus carnosus* by the identification method of 16S‐RNA molecular biology. Large amounts of LAB and yeast and several staphylococci were detected during fermentation of the low‐salt Suan rou. The color, flavor, and taste of Suan rou substantially improved under the synergistic effects of these dominant microorganisms.

The population of bacilli in the three batches of Suan rou amounted to 2.37 –2.47 log cfu/g at the initial fermentation. Thereafter, the level of bacilli notably decreased to 0 –0.43 log cfu/g during 30 days of fermentation. The results were similar to the those of Zeng et al. (2013a), who reported that the rapid growth of LAB may cause the suppressed growth of bacilli on fermented meat products.

In the fermented Suan rou samples, the initial numbers of Enterobacteriaceae, Enterococci, and Pseudomonades were 2.63–2.87 , 2.01–2.37, and 2.15 –2.35 log cfu/g, respectively. With mass reproduction of LAB and the reduction of product pH during fermentation, the numbers of these bacteria in the samples of batches B and C were below the limit of detection on the 30th day of fermentation, whereas the numbers of Enterobacteriaceae, Enterococci, and Pseudomonades in the batch A samples decreased to 0.67 ± 0.02, 0.53 ± 0.01, and 0.27 ± 0.01 log cfu/g on the 30th day of fermentation, respectively. This result indicates that compared with batch A, the growth of Enterobacteriaceae, Enterococci, and Pseudomonades, which are suitable for the reproduction of LAB, was inhibited in batches B and C. (Hu et al., 2008) also noted that high levels of LAB grown in silver carp surimi can notably inhibit the growth and reproduction of Enterobacteriaceae, Enterococci, and Pseudomonades. The high correlation (*r* = .903–.991, *p* < .05) between the pH and the number of Enterobacteriaceae, Enterococci, and Pseudomonades showed that pH is a key factor restraining the growth of other bacteria. (Lücke, 2000) speculated that the rapidly reduced pH and bacteriocin generated by LAB may be the main factors that restrain putrefying and pathogenic bacteria.

### 3.2 Physicochemical analysis

The nutritional value and flavor of fermented meat products is largely determined by the degradation degree of proteins in the samples. Compared with batch A, the contents of total, myofibrillar, and sarcoplasmic proteins in batches B and C showed higher protein degradation rates, which respectively decreased from 16.1% ± 0.49%,6.49% ± 0.19%, and 3.44% ± 0.12% to 10.1% ± 0.28%, 2.57% ± 0.09%, and 1.31% ± 0.06% in batch B and from 16.2% ± 0.49%, 6.49% ± 0.19%, and 3.43% ± 0.10% to 9.75% ± 0.27%,2.24% ± 0.09%, and 1.27% ± 0.06% in batch C with extended fermentation time(Table 1). A significant positive correlation was observed between the changes in total protein and myofibrillar and sarcoplasmic proteins (*r* = .983–.997, *p* < .01; *r* = .983–.994, *p* < .01, respectively). These results indicate that the proteolysis and amounts of small molecules increased significantly and showed certain nutritional significance during fermentation. The muscle proteins of Suan rou were severely hydrolyzed. This finding may be caused mainly by the endogenous cathepsin and microbial protease activity, which cause the formation of small peptides, FAAs, and ammonia N and contribute to the formation of aroma and flavor of fermented products due to protein degradation (Molly et al., 1997). Valyasevi and Rolle (2002) also reported that strong protease activity by endogenous cathepsin by *S. cerevisiae* and bacilli causes the breakdown of fish proteins into peptides and FAA sunder acidic environment, contributing to the unique flavor of the products.

**TABLE 1:** Changes of protein and lipid types during fermentation of Suan rou

Fermentation period, days	Protein type (%)			Lipid type		
	Protein	Myofibrillar	Sarcoplasmic	Fat (g/100 g)	Cholesterol (mg/100 g)	FFA (mg/10 0g)
0						
Batch A	16.3 ± 0.51^a^	6.47 ± 0.18^a^	3.42 ± 0.11^a^	35.6 ± 1.17^a^	97.3 ± 1.86^a^	0.34 ± 0.00^a^
Batch B	16.1 ± 0.49^a^	6.49 ± 0.19^a^	3.44 ± 0.12^a^	35.7 ± 1.18^a^	97.1 ± 1.83^a^	0.33 ± 0.00^a^
Batch C	16.2 ± 0.49^a^	6.49 ± 0.19^a^	3.43 ± 0.10^a^	35.6 ± 1.18^a^	97.1 ± 1.82^a^	0.33 ± 0.00^a^
5						
Batch A	15.5 ± 0.46^b^	5.93 ± 0.18^a^	3.11 ± 0.12^a^	34.8 ± 1.16^a^	96.1 ± 1.87^a^	0.43 ± 0.01^a^
Batch B	14.7 ± 0.41^a^	5.76 ± 0.15^a^	3.08 ± 0.11^a^	34.5 ± 1.14^a^	96.3 ± 1.88^a^	0.42 ± 0.01^a^
Batch C	14.3 ± 0.41^a^	5.71 ± 0.14^a^	3.05 ± 0.11^a^	34.3 ± 1.17^a^	96.4 ± 1.88^a^	0.43 ± 0.01^a^
10						
Batch A	14.7 ± 0.39^b^	5.74 ± 0.17 ^b^	2.93 ± 0.11^b^	33.7 ± 1.16^a^	92.1 ± 1.81^a^	0.71 ± 0.01^a^
Batch B	13.7 ± 0.33^a^	5.04 ± 0.15 ^a^	2.46 ± 0.10^a^	33.9 ± 1.17^a^	92.7 ± 1.82^a^	0.69 ± 0.01^a^
Batch C	13.5 ± 0.31^a^	5.01 ± 0.13^a^	2.42 ± 0.10^a^	33.4 ± 1.15^a^	91.7 ± 1.83^a^	0.69 ± 0.01^a^
15						
Batch A	14.2 ± 0.36^b^	4.95 ± 0.15^b^	2.37 ± 0.13^b^	33.1 ± 1.08^a^	88.7 ± 1.76^a^	0.97 ± 0.01^a^
Batch B	13.1 ± 0.33^a^	4.35 ± 0.14^a^	2.11 ± 0.12^a^	33.1 ± 1.07^a^	88.9 ± 1.78^a^	0.97 ± 0.01^a^
Batch C	12.7 ± 0.31^a^	4.28 ± 0.14^a^	2.08 ± 0.12^a^	33.2 ± 1.07^a^	88.9 ± 1.79^a^	0.96 ± 0.01^a^
20						
Batch A	13.8 ± 0.35^b^	4.27 ± 0.14^b^	2.12 ± 0.12^b^	31.8 ± 1.03^a^	87.5 ± 1.79^a^	1.18 ± 0.02^a^
Batch B	11.9 ± 0.31^a^	3.63 ± 0.12^a^	1.68 ± 0.09^a^	31.5 ± 1.01^a^	88.2 ± 1.81^a^	1.17 ± 0.02^a^
Batch C	11.4 ± 0.29^a^	3.52 ± 0.11^a^	1.62 ± 0.09^a^	31.6 ± 1.01^a^	87.1 ± 1.76^a^	1.16 ± 0.02^a^
25						
Batch A	13.1 ± 0.32^b^	3.94 ± 0.12^b^	1.83 ± 0.09^b^	31.1 ± 1.05^a^	83.7 ± 1.72^a^	1.31 ± 0.02^a^
Batch B	10.6 ± 0.28^a^	2.78 ± 0.11^a^	1.47 ± 0.07^a^	31.1 ± 1.03^a^	84.1 ± 1.78^a^	1.33 ± 0.02^a^
Batch C	10.1 ± 0.27^a^	2.64 ± 0.09^a^	1.41 ± 0.07^a^	31.3 ± 1.06^a^	83.5 ± 1.77^a^	1.32 ± 0.02^a^
30						
Batch A	12.5 ± 0.31^b^	3.53 ± 0.11^b^	1.57 ± 0.07^b^	30.6 ± 1.08^a^	81.3 ± 1.72^a^	1.61 ± 0.02^a^
Batch B	10.1 ± 0.28^a^	2.57 ± 0.09^a^	1.31 ± 0.06^a^	30.5 ± 1.07^a^	81.5 ± 1.81^a^	1.58 ± 0.02^a^
Batch C	9.75 ± 0.27^a^	2.24 ± 0.09^a^	1.27 ± 0.06^a^	30.5 ± 1.11^a^	80.7 ± 1.68^a^	1.62 ± 0.02^a^

Note

Batch A: Suan rou was prepared using Technique A; Batch B: Suan rou was prepared using Technique B; Batch C: Suan rou was prepared using Technique C. ^a,b,c^Values with unlike superscript letters in the same column are significantly different (*p* < .05) LSD test.

The contents of crude fat, cholesterol, and FFA in Suan rou showed no significant difference in fat content among the three groups (table 1). The levels of crude fat and cholesterol gradually decreased from 35.6 –35.7 and 97.1 –97.3 mg/100 g to 30.5 –30.6 and 80.7 –81.5 mg/100 g at the end of fermentation, respectively. Excessive intake of high fat and cholesterol diet is unhealthy and lacks flavor. Therefore, the reduction of crude fat and cholesterol caused by fermentation bears nutritional significance during Suan rou processing. In addition, the counts of FFA in the three batches reached 1.61 ± 0.02, 1.58 ± 0.02, and 1.62 ± 0.02 mg/100 g at the end of fermentation. The release of FFA plays an important role in providing healthy ingredients and flavor elements of the fermented meat product during ripening fermentation. A significant negative correlation was observed among the changes in crude fat, cholesterol, and FFA (*r *= – 989 to –0.994, *p* < .01(correlation coefficient of crude fat and cholesterol in technique A, B, C); *r* = –.983 to –0.993, *p* < .01(correlation coefficient of crude fat and FAA in technologies A, B, C);*r* = –.975 to –0.995, *p* < .01(correlation coefficient of cholesterol and FAA in technologies A, B, C), respectively). The amounts of yeast and *Staphylococcus* with lipase activity can slow the decomposition of fat and formation of FFAs. The counts of LAB were negatively correlated with crude fat and cholesterol levels (*r* = –.960 to –0.984, *p* < .01 (the range of correlation coefficients between lactic acid bacteria and crude fat and cholesterol in technologies A); *r* = –.874 to –0.889, *p* < .05(the range of correlation coefficients between lactic acid bacteria and crude fat and cholesterol in technologies B);*r* = –.888 to –.903, *p* < .05, (the range of correlation coefficients between lactic acid bacteria and crude fat and cholesterol in technologies C)respectively). Riebroy et al., 2008 reported that the combined action of acidophilous endogenous enzymes and microbial enzymes can cause the formation of FFAs and reduction in the levels of crude fat and cholesterol, thus contributing to the unique flavor and providing a health factor. In this study, we speculated that the reduction in crude fat and cholesterol and the production of FFAs may be the effect of various microorganisms, such as LAB and *Staphylococcus*, in Suan rou.

Reasonable aw in fermented food cannot only promotes the proliferation of favorable microorganisms and control the growth of unfavorable microbes but also change the textural parameters of products. In our research, the three groups of low‐salt fermented Suan rou showed similar aw (0.91 ± 0.04) (data not shown). According to the research of (Listner, 1975), a pH less than 5.2 and an aw less than 0.95 of fermented meat products are suitable for the extensive proliferation of LAB, and the products can be stored for a long term without cold storage. This result indicates that the aw of Suan rou plays an irreplaceable role in promoting the rapid acid production of the products, improving the safety and texture of the products, and restraining the growth of infectious bacteria.

**FIGURE 2:**
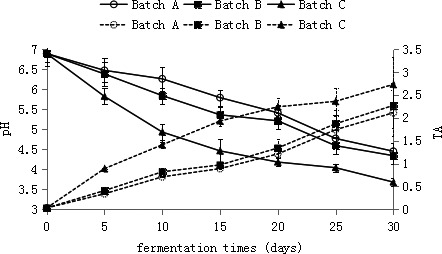
The values of pH and titratable acidity of the Suan rou samples during the ripening fermentation. TA, titratable acidity. Closed square, BatchA; Closed rhombus, BatchB; Closed Triangle, BatchC; Opened square, BatchA; Opened Rhombus, BatchB;Opened Triangle, BatchC; the solid line represents pH, and the dashed line represents TA

Figure 2 shows the pH and TA of Suan rou during fermentation. The figure shows that compared with the batch A sample, the samples in batches B and C have shown a faster pH reduction speed and a higher TA generation amount (*p* < .05). The pH reduced from 6.89 ± 0.06 to 4.34 ± 0.04–3.68 ± 0.03 in batches B and C at the end of fermentation, and the TA values increased from 0.03 ± 0.00 g/kg to 2.27 ± 0.05 and 2.73 ± 0. 5 g/kg, respectively. The Suan rou in batch A showed a slow acid production speed. The pH of batch A reduced to 4.45 ± 0.06, whereas the TA increased to 2.11 ± 0. 5g/kg at the end of fermentation. In the samples, pH exhibited a high negative correlation with TA (*r *= –.985 to –.995, *p* < .01). The rapid generation of LAB in the fermented meat products is strongly related to the reduction of pH value and rise of TA, whereas the fast proliferation of LAB is closely related to nutrient substances, such as glucose and saccharose, added to the product. These saccharides can provide energy for the rapid propagation of LAB. Saccharose was added to the Suan rou of batch B during processing, thus notably promoting the rapid generation of LAB. The 2% salt concentration can promote the rapid proliferation of LAB. Normally, LAB can grow under 4%–5% salt, but the growth rate will be partially restrained. The salt content added in the Suan rou of batch A during the processing period was between 4% and 5%, which resulted in the low reduction rate of pH and the low generation rate of TA. According to the research of (Riebroy et al., 2004), organic acids such as lactic acid that accumulated in fermented meat products result in low pH and high TA of the products and are an essential factor controlling the growth of other putrefying and pathogenic bacteria, implying the high safety of the products. Furthermore, with the reduction of pH and accumulation of organic acids, the product texture will also be influenced, resulting in the increased hardness and enhanced cohesive force and elasticity.

Organic acid content is also an important factor guaranteeing product safety. In raw meat, lactic and acetic acids are both below the limits of detection. Lactic acids increased to 1.76% ± 0.01%, 2.21% ± 0. 01%, and 2.67% ± 0. 01% in batches A, B, and C at the end of fermentation, respectively (data are not shown). Compared with batch A, batch B, which was added with saccharose, and batch C, which was added with 2% salt, presented higher lactic acid generation amounts (*p* < .05). Meanwhile, 0.11% –0.12% acetic acid (*p* > .05) was detected in the three different low‐salt fermented Suan rou. This finding indicates that lactic acid is the main organic acid generated in fermentation, whereas a small amount of acetic acid is also generated. Meanwhile, oxalic, succinic, and butyric acids were below the limits of detection. These research results are similar to those of other reports on organic acids in fermented meat products. For example, the lactic acid generated by the reproduction and metabolism of LAB becomes the main organic acid in the fermented meat products of Som‐fug and Salchichón (Riebroy et al., 2004). (Ostergaard et al., 1998) reported that in fermented meat products, 2.2%–2.5% of lactic acid causes the high sensory quality of the product. For this reason, proper lactic acid and acetic acid contents in Suan rou can satisfy the traditional product requirements, namely, good taste and flavor, of consumers. Acid accumulation reduces the product pH to near the isoelectric point of proteins, which can promote the dehydration and reduce the Aw of the product.

### 3.3 SDS‐PAGE analysis

**FIGURE 3:**
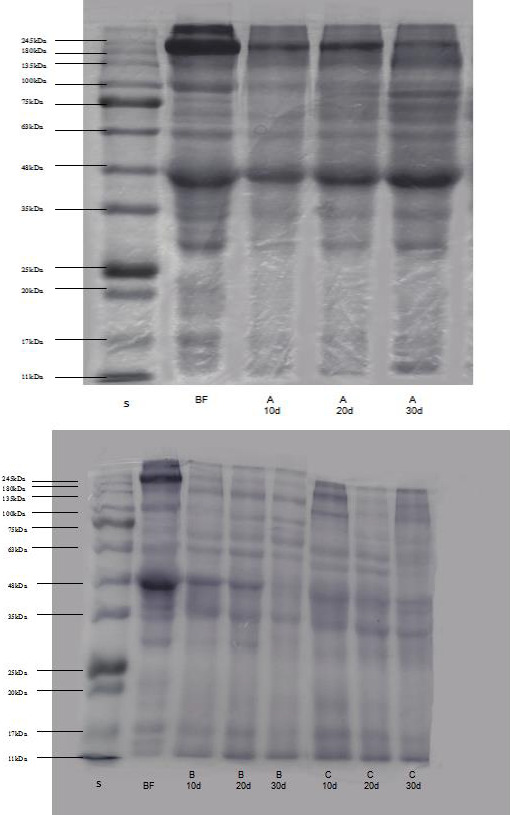
Changes in SDS‐PAGE profile of muscle proteins in Suan rou samples. S: protein markers; BF: before fermentation

Figure 3 displays the electrophoretic profiles of muscle proteins in the three samples and raw material. Among the three samples evaluated, SDS‐PAGE gel lanes showed distinct changes in banding patterns in response to the raw material. The majority of muscle protein extract resulted in the marked decrease in major bands and the appearance of polypeptides with molecular weights less than 20 kDa. The protein bands with sizes of approximately 245, 135, 100, 63, 48, 35, and 20 kDa decreased. The bands at 11 kDa were more intense in the three samples compared with the raw material. During ripening fermentation, the polypeptides with molecular weights of 245, 135, 63, 48, and 35 kDa showed a more intense decrease in batches B and C compared with batch A. Furthermore, the intensity of the 48 kDa band severely decreased at the end of fermentation. Zeng et al. (2013a) detected a considerable muscle protein degradation during ripening fermentation. The proteolysis in Suan rou is probably affected by elements, including product formulation, indigenous microorganisms, and processing technology, due to the action of proteinases from *Staphylococcus* or LAB and the hydrolysis of muscle proteins caused by acids (Astiasarán et al., 1990). Therefore, in our study, the differences in microorganism distribution, acidity, and endogenous enzyme activity caused by the different amounts of sodium chloride and sucrose in the samples may cause the varying degrees of hydrolysis of the Suan rou protein in the different groups, whereas intense partial bands were observed due to the possible co‐migration of hydrolysis from large proteins.

### 3.4 FAA analysis

FAA can improve product flavor. Thus, the release of FAA during the fermentation of meat product must be investigated. Table 2 lists the FAA change conditions of the three different groups of low‐salt fermented Suan rou. The table shows that compared with the fresh streaky pork before fermentation (BF), the species and quantity of FAAs in the three groups of fermented Suan rou have evidently increased. Batches B and C showed a faster generation rate (*p* < .05) of FAAs than batch A. However, no evident distinction was observed between the type and amount of the final products in the three groups of Suan rou (*p* > .05). In the final products of fermented Suan rou, high amounts of essential amino acids (EAAs) for the human body, such as lysine, leucine, and glycine, were detected. The total amount of FAAs increased from 163 ± 3.61 mg/100 g to 199 ± 5.34, 213 ± 5.79, and 209 ± 5.62 mg/100 g. The evident growth rates of glycine, glutamic acid, aspartic acid, valine, leucine, lysine, and arginine were noted. In the determined FAAs, the content of glutamic acid (35.6 ± 0.27–39.4 ± 0.27 mg/100 g) was the highest at the last stage of fermentation. Compared with the results in existing reports on fermented meat products, the species and quantity of FAAs in Suan rou were notably higher than those in other fermented meat products. According to the research of (Mukherjee et al., 2014), the generation of FAAs in fermented meat products results from the fermentation and maturity process; cathepsin shows high activity at pH between 5.0 and 5.5; thus, the proteins are degraded, and the protease of microorganisms (LAB, staphylococci, and yeast) further accelerates the degradation of muscle proteins during fermentation (Qiu et al., 2012). We speculate that the rapid generation rate of the three types of bacteria in fermented Suan rou may result in the rapid generation rate of FAAs in the Suan rou of the two groups. Existing research indicates that the content of FAAs shows a high correlation with the flavor of fermented products. Consequently, the highly concentrated FAAs generated in fermentation can contribute to the formation of unique product flavors. The species and content of total amino acids in batches A, B, and C were higher than those in the batch BF. This finding was observed possibly because during Suan rou fermentation, the enzymes secreted by yeasts and LAB can partly degrade molecular proteins into flavorful substances, such as small molecular peptides and FAAs. In this research, EAA and *delicious* *amino acid* contents in batches A, B, and C showed improvement compared with those in the BF batch (*p* < .05), thus largely contributing to Suan rou's nutrient and flavor formation.

**TABLE 2:** Changes in free amino acids of Suan rou during fermentation

free amino acid (mg/g)	Sample			Batch C
	BF	Batch A	Batch B	
Asp	17.1 ± 0.22^a^	21.3 ± 0.23^b^	23.7 ± 0.25^c^	23.4 ± 0.25^c^
Thr	8.37 ± 0.16 ^a^	10.6 ± 0.16 ^b^	11.4 ± 0.17^b^	11.1 ± 0.16 ^b^
Ser	7.34 ± 0.17 ^a^	8.61 ± 0.18 ^b^	8.51 ± 0.18 ^b^	8.62 ± 0.17 ^b^
Glu	29.7 ± 0.26 ^a^	35.6 ± 0.27^b^	39.4 ± 0.27 ^c^	36.8 ± 0.26 ^b^
Gly	7.32 ± 0.14 ^a^	8.58 ± 0.15 ^c^	8.86 ± 0.14^b^	8.87 ± 0.16 ^b^
Ala	1.21 ± 0.02 ^a^	1.54 ± 0.02 ^b^	1.68 ± 0.02 ^c^	1.63 ± 0.03 ^c^
Cys	1.16 ± 0.03 ^a^	1.59 ± 0.03 ^b^	1.72 ± 0.03 ^c^	1.71 ± 0.02^c^
Val	9.15 ± 0.19 ^a^	11.3 ± 0.18 ^b^	12.4 ± 0.16^d^	11.8 ± 0.17 ^c^
Met	2.37 ± 0.07 ^a^	3.18 ± 0.09 ^b^	3.14 ± 0.06^b^	3.15 ± 0.03 ^b^
Ile	9.16 ± 0.17 ^a^	11.8 ± 0.14 ^b^	12.7 ± 0.18 ^c^	12.5 ± 0.17^c^
Leu	16.8 ± 0.26 ^a^	20.7 ± 0.21^b^	23.8 ± 0.27 ^c^	24.7 ± 0.27 ^d^
Tyr	9.31 ± 0.18^a^	10.6 ± 0.17 ^b^	11.2 ± 0.17^c^	11.1 ± 0.16 ^c^
Phe	6.33 ± 0.11 ^a^	7.26 ± 0.13^b^	7.07 ± 0.09^b^	7.18 ± 0.11^b^
Lys	12.3 ± 0.17 ^a^	15.7 ± 0.21^b^	16.9 ± 0.24 ^c^	15.9 ± 0.18^b^
His	9.36 ± 0.16 ^a^	9.81 ± 0.17 ^b^	9.67 ± 0.15 ^b^	9.73 ± 0.07 ^b^
Arg	12. 3 ± 0.18 ^a^	15.5 ± 0.21 ^b^	15.6 ± 0.21^b^	15.3 ± 0.19^b^
Pro	3.86 ± 0.07 ^a^	4.95 ± 0.06^b^	5.61 ± 0.07 ^c^	5.63 ± 0.08 ^c^
TAA	163 ± 3.61 ^a^	199 ± 5.34^b^	213 ± 5.79^c^	209 ± 5.62^c^
EAA	16.5 ± 0.86^a^	20.8 ± 0.93^b^	20.3 ± 0.87^b^	19.5 ± 0.79^b^
DAA	19.1 ± 0.76^a^	24.1 ± 1.33^b^	23.4 ± 1.16^b^	22.6 ± 1.19^b^
NEAA	23.9 ± 0.96^a^	36.2 ± 1.41^c^	33.7 ± 1.45^bc^	32.5 ± 1.51^b^
EAA/TAA (%)	40.9 ± 1.53 ^b^	36.5 ± 1.21 ^a^	37.1 ± 1.32 ^a^	37.4 ± 1.31 ^a^
DAA/TAA (%)	47.3 ± 1.62 ^b^	42.3 ± 1.26 ^a^	42.7 ± 1.29 ^a^	43.3 ± 1.34 ^a^
EAA/DAA	0.86 ± 0.03	0.86 ± 0.03	0.87 ± 0.03	0.86 ± 0.03

Note

Batch A: Suan rou was prepared using Technique A; Batch B: Suan rou was prepared using Technique B; Batch C: Suan rou was prepared using Technique C. ^a,b,c^ Values with unlike superscript letters in the same row are significantly different (*p* < .05) LSD test.

Abbreviations: AA, delicious amino acid; BF, before fermentation; EAA, essential amino acid; NEAA, non‐essential amino acid; TAA, total amino acids.

### 3.5 FA analysis

Fatty acid degradation plays an important role in the formation of flavor of fermented meat products in the fermentation and ripening process of meat products. In most cases, the release of FFAs occurs as a series of biochemical reactions under the combined action of enzymes of meat products or microorganic lipases, which will form the unique flavor substance of the fermented product (Casaburi et al., 2007). Table 3 shows the composition of saturated, monounsaturated, and polyunsaturated fatty acids in the fermentation of the three different groups of low‐salt Suan rou. The main fatty acids in the samples BF included oleic (49.1 ± 0.79 mg/g), palmitic (15.2 ± 0.23 mg/g), stearic (9.27 ± 0.12 mg/g), and linolenic acids (14.8 ± 0.16 mg/g). The content of saturated fatty acid in the samples BF was the highest. However, with prolonged fermentation, the content percentages of these acids in the three groups of Suan rou samples gradually decreased, wherein the decrease in oleic acid content was the most evident. In the Suan rou samples at the last stage of fermentation, the main FFAs included palmitic, oleic, myristic, palmitoleic, linoleic, and linolenic acids. The content percentage of polyunsaturated fatty acids considerably increased, especially the ω‐3 and ω‐6 unsaturated fatty acids, which are functional in the human body. Similar conclusions can be found in the work of (Riebroy et al., 2008) on FFAs of Som‐fug; the content of saturated fatty acids in Som‐fug during fermentation notably decreased, and high amounts of polyunsaturated fatty acids, such as linoleic, linolenic, docosahexaenoic, and eicosapentaenoic acid, were formed. Verplaetse considered that the increased FFAs may be the result of the combined action of acidophilous endogenous enzymes and microbial enzymes in meat products.

**TABLE 3:** Changes in fatty acids of Suan rou during fermentation

fatty acids (mg/g)	Sample			Batch C
	BF	Batch A	Batch B	
C12:0 (Lauric acid)	0.89 ± 0.01^a^	1.12 ± 0.02^c^	1.07 ± 0.02^b^	1.15 ± 0.02^c^
C14:0 (Myristic acid)	4.17 ± 0.08^a^	5.21 ± 0.08^b^	5.43 ± 0.07^c^	5.36 ± 0.08^bc^
C16:0 (Palmitic acid)	15.2 ± 0.23 ^a^	15.5 ± 0.21 ^a^	15. 3 ± 0.22^a^	15. 6 ± 0.24 ^a^
C17:0 (Margaric acid)	0.12 ± 0.00^a^	0.13 ± 0.01^a^	0.13 ± 0.01^a^	0.12 ± 0.01^a^
C18:0 (Stearic acid)	9.27 ± 0.12 ^b^	9.38 ± 0.11^b^	8.81 ± 0.12^a^	9.67 ± 0.13^b^
C20:0 (Arachidic acid)	1.31 ± 0.02^b^	1.28 ± 0.02 ^ab^	1.24 ± 0.01^a^	1.26 ± 0.03 ^a^
SFA	30.1 ± 0.62^a^	33.4 ± 0.69^b^	31.7 ± 0.71^a^	32.3 ± 0.73^ab^
C14:1 (Oleum cardamomi acid)	0.14 ± 0.00^a^	1.34 ± 0.02^a^	1.42 ± 0.03^a^	1.37 ± 0.01^a^
C17:1 (Heptadecanoic acid)	0.09 ± 0.00 ^a^	0.10 ± 0.00 ^b^	0.11 ± 0.01^b^	0.09 ± 0.00^a^
C18:1 (Oleic acid)	49.1 ± 0.79^b^	44.8 ± 0.68 ^a^	43.9 ± 0.86^a^	46.3 ± 0.77 ^a^
MUFA	50.2 ± 0.86^c^	45.7 ± 0.69^a^	46.1 ± 0.64^ab^	47.2 ± 0.62^b^
C18:2 (Linoleic acid)	3.16 ± 0.02 ^a^	3.87 ± 0.02 ^c^	3.71 ± 0.02 ^b^	3.68 ± 0.02^b^
C18:3 (Linolenic acid)	14.8 ± 0.16 ^a^	14.7 ± 0.11^a^	14.8 ± 0.17^a^	14.7 ± 0.17 ^a^
C20:4 (Arachidonic acid)	1.33 ± 0.02 ^a^	1.61 ± 0.03^b^	1.67 ± 0.03^b^	1.68 ± 0.03^b^
C20:5 (Eicosapentaenoic acid)	0.13 ± 0.00 ^a^	0.19 ± 0.00^b^	0.19 ± 0.00^b^	0.18 ± 0.00^b^
C22:6 (Docosahexaenoic acid)	0.29 ± 0.01 ^a^	0.87 ± 0.02 ^b^	0.94 ± 0.02^c^	0.99 ± 0.03 ^d^
PUFA	20.8 ± 0.26^a^	22.1 ± 0.24^b^	20.4 ± 0.23^a^	21.7 ± 0.25^b^
SFA:MUFA:PUFA	1:1.6:0.7	1:1.4:0.7	1:1.4:0.6	1:1.5:0.7

Note

Batch A: Suan rou was prepared using Technique A; Batch B: Suan rou was prepared using Technique B; Batch C: Suan rou was prepared using Technique C. ^a,b,c^Values with unlike superscript letters in the same row are significantly different (*p* < .05) LSD test.

Abbreviations: BF, before fermentation; MUFA, monounsaturated fatty acid; PUFA, polyunsaturated fatty acids; SFA, saturated fatty acid.

### 3.6 TVB‐N analysis

**FIGURE 4:**
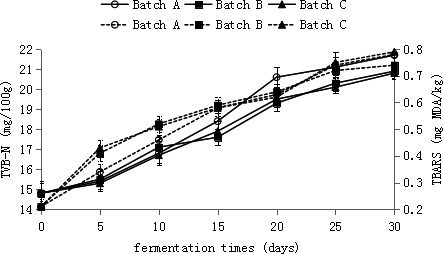
Changes in TVB‐N values, TBARS values in Suan rou samples during the ripening fermentation. The solid line represents TVB‐N, the dashed line represents TBARS. Closed square, BatchA; Closed rhombus, BatchB; Closed Triangle, BatchC; Opened square, BatchA; Opened Rhombus, BatchB; Opened Triangle, BatchC

TVB‐N is a traditional chemical component index for expressing the decaying degree of meat products. The decarboxylation of amino acids and the reproduction of putrefying bacteria in food can result in the formation of TVB‐N. In our research on each group of Suan rou, TVB‐N in the Suan rou of batches B and C increased from 14.8 ± 0.03 mg/100 g to 20.9 ± 0.04 and 20.8 ± 0.03 mg/100 g at the end of fermentation, respectively (Figure 4). The TVB‐*N* values in the two groups of samples showed no notable increase (*p* > .05), whereas that in the Suan rou of batch A increased to 21.7 ± 0.05 mg/100 g at the last stage of fermentation. Thus, the TVB‐*N* values of batches B and C, which presented rapid lactic acid generation rates, were evidently lower than that of batch A.

The TVB‐*N* values of batches B and C showed no sharp improvement. This result is possibly due to the high amounts of lactic acids generated by fermentation of LAB, resulting in acid–base neutralization. The TVB‐*N* value was within a controlled range; thus, the freshness of Suan rou can be maintained to a great extent. (Hu et al., 2008) reported that in the fermented silver carp surimi, the group inoculated with a leavening agent can restrain the accumulation of TVB‐N. In this research, the TVB‐*N* values for all fermented Suan rou have not exceeded the standard formulated by European Union, that is, the TVB‐*N* value should not be higher than 35 mg TVB‐N 100 g^‐1^ in fish. This finding indicates that the three types of low‐salt fermented Suan rou all exhibited low TVB‐*N* values.

### 3.7 Fat oxidation analysis

Thiobarbituric acid reactive substance (TBARS) value has been widely used as an index to predict the fat oxidation degree of meat and meat products. According to previous research, the maximum TBARS value is 5 mg *malondialdehyde* (MDA)/kg to ensure the high quality of meat (Sikorski et al., 1990). In our research, the initial TBARS for each Suan rou sample was between 0.21 ± 0.02 mg MDA/kg. After 30 days of fermentation, the TBARS value of batch A has reached 0.78 ± 0.05 mg MDA/kg, whereas those of batches B and C have increased slowly given that LAB can be rapidly generated in batches B and C. After 30 days of fermentation, the TBARS values of batches B and C respectively increased to 0.74 ± 0.05 and 0.79 ± 0.05 mg MDA/kg (Figure 4). The TBARS value of batch A was significantly higher than that of batch B (*p* < .05), and no distinct difference was observed between the TBARS values for batch B and C during fermentation (*p* > .05) (Hu et al., 2008) reached similar conclusions on the fermented surimi sausage, which was inoculated with LAB and *Staphylococcus*. We speculate that the reason for the lower detection value of TBA in batch B may be the effect of antioxidant active substances produced by fast‐proliferating LAB and Staphylococcus on the oxidation of unsaturated fatty acids. Such as: superoxide dismutase (SOD), catalase (CAT) and other antioxidant enzymes.

### 3.8 Textual analysis

Table 4 lists the hardness, springiness, resilience, chewiness, gumminess, and cohesiveness of Suan rou during fermentation. The hardness and chewiness of Suan rou remarkably increased (*p* < .05), whereas no distinct difference was observed between resilience (*p* > .05) at the end of fermentation.

**TABLE 4:** Texture profile analysis for Suan rou

Parameters	Sample			
	BF	Batch A	Batch B	Batch C
Hardness (g)	539 ± 26.4 ^a^	718 ± 38.4 ^b^	786 ± 41.3 ^b^	726 ± 31.7^b^
Springiness (mm)	0.92 ± 0.03^d^	0.84 ± 0.03 ^c^	0.78 ± 0.02 ^b^	0.71 ± 0.02 ^a^
Cohesiveness	0.83 ± 0.03^c^	0.59 ± 0.03 ^a^	0.61 ± 0.02^a^	0.71 ± 0.03 ^b^
Gumminess (g)	236 ± 11.4 ^a^	427 ± 16.7 ^b^	438 ± 18.3 ^b^	429 ± 17.8 ^b^
Chewiness (g mm)	186 ± 7.46 ^a^	337 ± 12.4 ^b^	348 ± 13.6^b^	335 ± 12.5 ^b^
Resilience	0.10 ± 0.01 ^a^	0.12 ± 0.01^a^	0.12 ± 0.01^a^	0.12 ± 0.01 ^a^

Note

Batch A: Suan rou was prepared using Technique A; Batch B: Suan rou was prepared using Technique B; Batch C: Suan rou was prepared using Technique C. ^a,b,c,d^Values with unlike superscript letters in the same row are significantly different (*p* < .05) LSD test.

Abbreviation: BF, before fermentation;

With the decreased pH and Aw, the hardness and chewiness of the three different types of low‐salt fermented Suan rou evidently strengthened (*p* < .05) compared with those BF. In batches B and C, which presented faster lactic acid generation rate and pH decrease rate, the hardness and chewiness of the products exhibited more evident increase than those in batch A during the ripening fermentation. No significant difference was detected in the hardness and chewiness of the final products of the three groups of Suan rou (*p* > .05). In the fermentation of meat products, the decrease in pH close to the isoelectric point of proteins causes the partial dehydration of meat products, induces the gathering of myofibrillar proteins in fermented meat products, and results in acid‐induced gel formation (Xu et al., 2012). We speculate that the coordinating influence of these factors has resulted in good hardness and chewiness of the final products of fermentation. (Riebroy et al., 2008) also shared similar research on Som‐fug and indicated the notably increased hardness and chewiness of Som‐fug.

Cohesiveness can embody the strength of intermolecular forces in fermented meat products. The research of (de Huidobro et al., 2005) indicates that for fermented meat products, a pH below 5.0 implies a classic negative correlation with cohesiveness. The present research has demonstrated that in low‐acid‐fermented meat products, relevant products showed relatively low cohesive forces given that the moderate‐strength acid environment reduced the isoelectric point of proteins. Suan rou is a type of acid‐fermented meat product with a pH lower than 4.5. In the fermentation of Suan rou, the pH during part of the fermentation time periods is close to the isoelectric point of protein, resulting in the denaturing of protein gel and therefore the strengthening of intermolecular cohesive force. On the 5th–10th day of fermentation of Suan rou in batches B and C and the 15th–20th day of fermentation in batch A, a high cohesiveness was observed (data are not shown), whereas in the initial and last stages of fermentation, the pH was evidently higher or lower than 5.0. Thus, the cohesive forces of Suan rou in the earlier and later stages of fermentation are relatively low.

### 3.9 Sensory analysis

**FIGURE 5:**
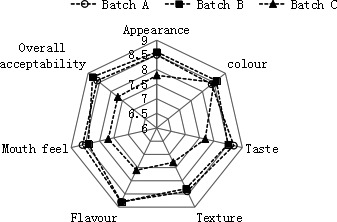
Sensory characteristics in Suan rou samples. Closed square, BatchA; Closed rhombus, BatchB; Closed Triangle, BatchC

Figure 5 shows the sensory evaluation results of three different low‐salt fermented Suan rou. The figure shows that the Suan rou of batches A and B is superior to that of batch C in terms of appearance, flavor, taste, mouth feel, and overall acceptability (*p* < .05). Although the Suan rou of batch C exhibited a faster fermentation speed, its taste was more acidic than those of batches A and B. The coordination between the acidity and salinity of Suan rou is the main factor influencing the acceptance of consumers for this traditional product. (Ostergaard et al., 1998) indicated that consumers would feature a high recognition degree toward low‐salt, acid‐fermented meat products only when the lactic acid concentration reaches at least 2%, the salt concentration range is between 2.5% and 4.5%, and the pH is lower than 4.5. Acids were rapidly generated in batch C, and the salt content was 2.13% ± 0.04%. The coordination between the acidity and salinity was reasonable in batches B and A, thus influencing the sensory quality of the product. The hydrolyzation of muscle proteins and fats can also improve the flavor of fermented products. The sensory analysis on fermented silver carp surimi conducted by (Hu et al., 2008) also indicated that high‐concentrated small molecular peptides, amino acids, aldehydes, and organic acids can notably improve the acceptability of fermented meat products. Consequently, the high acidity generated in the fermentation process, low salt, hydrolyzation of muscle proteins, and the degradation of fats form the unique flavor of Suan rou.

## 4 CONCLUSION

Suan rou is a traditional fermented meat product. Compared with those in other similar fermented meat products, the LAB (mainly including *L. plantarum* and *P. pentosaceus*) and saccharomycetes (mainly including *S. cerevisiae* and *Hansenula polymorpha*) in Suan rou rapidly reproduced during the whole fermentation, and substantial amounts of organic acids such as lactic acid were generated. Batch B, which was added with saccharose, and batch C, which contained a low‐salt concentration, exhibited a faster pH reduction rate and TA generation rate compared with batch A, therefore forming the unique and soft sour flavor of Suan rou. Compared with other fermented meat products, Suan rou also features better textual parameters, such as hardness and chewiness. Suan rou contains high levels of FFAs and FAAs that provide this product with rich nutrition and flavor. The sensory analysis of Suan rou indicated that 2% salt addition may result in bad flavor due to high acidity, whereas 4% salt addition amount in batch A and 2% saccharose addition in batch B resulted in a unique fermentation flavor favored by consumers. Combined with the overall indicators, the Suan rou produced by B technology is more easily accepted and loved, and can be used in industrial production.
